# Is domestic polyester suitable for plastination of thin brain slices?

**DOI:** 10.1590/1414-431X2023e12566

**Published:** 2023-06-30

**Authors:** L.S. Juvenato, Y.F. Monteiro, R.P. Miranda, A.P.S.V. Bittencourt, A.S. Bittencourt

**Affiliations:** 1 Laboratório de Plastinação, Departamento de Morfologia, Centro de Ciências da Saúde, Universidade Federal do Espírito Santo, Vitória, ES, Brasil; 2 Programa de Pós-Graduação em Bioquímica, Universidade Federal do Espírito Santo, Vitória, ES, Brasil; 3 Departamento de Ciências Fisiológicas, Universidade Federal do Espírito Santo, Vitória, ES, Brasil

**Keywords:** Brain, Plastination, Unsaturated polyester, Nervous tissue, Brazilian marketing, Domestic polyester

## Abstract

Plastination is a technique used to preserve biological tissues while retaining most of their original appearance. In the technique, developed by Dr. Gunther von Hagens in 1977, specimens were impregnated with a polymer, such as silicone, epoxy, or polyester. Considered the most suitable material for brain plastination, polyester has a wide application in teaching and research compared with imaging techniques. The materials for plastination are usually imported from Germany and more expensive than domestic products. If domestic polymers were to enter the market it would favor the expansion of plastination in Brazil. Hence, this study evaluated the feasibility of using domestic polyesters to replace the usual Biodur^®^ (P40) in plastination of brain slices. For this evaluation, 2-mm-thick sections of bovine brains were prepared and plastinated with domestic polyester. Slices were compared before impregnation and after curing using standardized photographs taken after dehydration and after curing. Plastination followed the standard protocol: fixation, dehydration, forced impregnation, and curing. Fifteen brain slices were plastinated with each polyester (P40, P18, and C1-3). There was no significant difference in the percent shrinkage between groups after plastination of P18 and P40, but the curing time of Cristalan^©^ polymer was too short for impregnation. Therefore, no initiator was used for C polymers impregnation. Thus, domestic polyester P18 was a viable option for the process.

## Introduction

Using biological materials for teaching, research, and university extension requires stabilization to preserve tissue structure and avoid natural decomposition. Over time, several substances have been discovered and developed for tissue fixation and conservation, such as heavy salts, tannins, glycerin, alcohols, phenols, and aldehydes (1,2).

Discovered in 1867 by German chemist August Wilhelm von Hofmann, formaldehyde (methanal) became the fixation and preservation icon for biological tissues and anatomical specimens and is commonly used in morphology laboratories due to its low cost, rapid tissue penetration, and long preservation capacity (3). It is highly toxic, carcinogenic (teratogenic), and highly irritating to mucous membranes, posing an immediate occupational risk to students, faculty, and technicians (2).

In the search for a substitute for formaldehyde conservation, a new technique (plastination) emerged in the late 1970s. Plastinated specimens are odorless, moisture-free, durable, non-toxic, maintenance-free (4,5), and prevent students, technicians, and faculty from coming into contact with formaldehyde (6).

Plastination is a process in which body fluids and fat are replaced by a polymerizable resin. According to von Hagens et al. (7), plastination involves four steps: fixation in formalin, acetone dehydration, forced impregnation with a curable polymer, and polymerization. Silicone, polyester, or epoxy are the main classes of polymers used (8). Silicone is used to preserve organs and whole specimens, whereas epoxy and polyester are used for serial sections (2-5 mm thick). Polyester is more suitable for nerve tissues, as it allows a greater differentiation between white and gray matter.

Worldwide, polyester plastination mainly uses the P40 resin from the German company Biodur (9,10), as it was developed and evaluated for this specific use. As for curing, P40 differs from other plastination polymers in that it is polymerized with ultraviolet light since a photo initiator has been added to the formulation. P40 is a relatively expensive product, especially in Brazil, because of transport and import duties. Thus, research on alternative resins for national marketing will help to disseminate the technique, reduce the cost of materials and promote it as a tool for teaching and applied research. Moreover, the development of this type of study will help it to be replicated with other resins and/or by other researchers.

Hence, this study evaluated the feasibility of using Brazilian polyesters to replace the Biodur^®^ P40 polyester in slice plastination.

## Material and Methods

Domestic polyester resins available in Brazil were obtained and technical leaflets were evaluated to assure the minimal requirements for plastination were listed: polymerization time, transparency, purity, and viscosity. Four resins with chemical catalyst curing, high transparency and purity, and low viscosity (<900 mPa.s) were selected for testing: 3 Cristalans (C1, C2, and C3) from Novapol^®^ and Arazyn 1.0 #08 (P18) from Redelease^®^. The viscosity range (<900 mPa.s) was selected according to studies on the relationship between polymer viscosity and tissue shrinkage (2). These resins are used for floor coating, construction, and in arts and crafts, but have never been evaluated for conservation of biological tissues. The characteristics of each polymer are summarized in Table 1.

**Table 1:** Basic characteristics listed on the data sheets of the selected polyesters.

Resin	Characterization	Solvents	Dynamic viscosity (cP)
P40	No specification on the data sheet	Styrene and benzyl methacrylate	33
C1, C2, and C3	Unsaturated, orthophthalic polyester resin	Styrene	300, 600, and 825, respectively.
P18	Unsaturated, orthophthalic polyester resin	Styrene	170-210

Unlike P40 resin, which is polymerized by ultraviolet light, the domestic polymers can be cured by adding a chemical initiator. Manufacturers suggest using 1% (v/v) of the initiator for a 30-min complete curing time. The resin and initiator start a chemical reaction, and the liquid resin begins to increase in viscosity entering a gel phase within 20-25 min, which is too viscous for routine impregnation. Because this time is too short for a satisfactory impregnation (10,11), it was necessary to decrease the percent of initiator to be mixed with the resin to allow enough time for complete impregnation and hardening. A curing time of 48-72 h was assumed to be appropriate and sufficient for impregnation, filling of the flat chambers for the curing phase, and for eventual procedural delays.

Polymerization begins as soon as the initiator is mixed with the resin, with a gradual and slow increase in viscosity until the moment preceding the gel state in the final period of curing, when viscosity increases at exponential rates (inflection point of the viscosity *vs* time curve) (2). To establish the amount of initiator necessary to allow the impregnation mix to stay fluid long enough for impregnation yet cure in a reasonable time, 5×50 mL aliquots of each polyester were well-mixed with descending concentrations [1, 0.5, 0.25, 0.125, and 0.0625% mass/mass (m/m)] of Butanox^®^ (Redelease, Brazil) initiator and allowed to cure to confirm polymerization time.

Results were recorded and examined to determine which concentration of initiator and polyester remained fluid for >2 days but <3 days (enough time for impregnation and polymerization). Results indicated that only P18 with ≤0.125% of initiator had the potential to allow complete impregnation. The short curing time of all the “C resin mixes”, even with small amounts of initiator, were not acceptable for impregnation.

Five bovine heads, donated by the Mafrical meat-packaging facility, located in Cariacica, Espírito Santo, Brazil were used for this study. The research was approved by the Animal Ethics Committee of the Federal University of Espírito Santo (CEUA-UFES), under No. 31/2019. Immediately after receiving the heads, they were opened with the aid of a circular saw and the brains were carefully removed. The specimens were washed under running water to remove blood and clots, fixed by weekly immersion in formalin baths with increasing concentrations of 2, 5, 7, and 10%, and refrigerated (5-7°C). Brains were stored in 10% formalin for at least 5 months to ensure complete fixation (12).

After thorough fixation, the brains were sectioned with a Bermar^®^ (model BM 07 NR, Brazil) conventional deli meat slicer, set at 2-mm cutting thickness (13). To facilitate storage and handling, the 75 slices were randomly organized into groups of 5 slices each. The slices were separated from each other by a cotton mesh and a plastic mesh/grid (size: 15x15 cm, holes: 7x7 mm, Darice^®^, Brazil). Finally, they were protected/contained with custom-made wire mesh (size: 15x15 cm, holes: 5x5 mm) around the perimeter and each group of 5 was secured with string, forming a “sandwich”. Dehydration of the sections (tied groups of 5) was performed with four consecutive cold (-25°C) weekly acetone baths of 95, 95, 100, and 100%, inside a freezer. The grids containing the specimens were positioned vertically during dehydration to prevent the slices from weighing each other down and to facilitate the escape of acetone bubbles (10,14).

The amount of acetone used in each immersion bath was 10:1 (v/v) ratio of acetone to biological material (15). Dehydration was complete when the acetone was greater than 99% (v/v) pure after the last bath, as measured with an acetonometer (5). To standardize the dehydration step, all sections were dehydrated together in the same container.

After dehydration, the sections were randomly distributed into five experimental groups defined by the polyester manufacturer: Redelease (P18 polyester), Biodur (P40 polyester), and Novapol (C1, C2, and C3) (Table 2). This step was performed in triplicate, with five specimens in each of the five polyesters, totaling 25 specimens per vacuum run and 15 specimens per polyester group.

**Table 2:** Experimental impregnation of tested polyesters.

Manufacturer	Resin/Experimental group
Biodur^®^	P40
Redelease^®^	P18
Novapol^®^	C1
	C2
	C3

Each group of 5 dehydrated slices was removed from the acetone and submerged into one of the five polyester containers in the vacuum chamber. For the P18 polyester, 0.125% (w/w) Butanox initiator was added with a micropipette as part of the impregnation mixture. For resins C1, C2, and C3 and for P40 polyester, no initiator was added (Table 3). The results of initiator concentration as a function of curing time showed that mixing the initiator at the beginning of forced impregnation would be impractical (Table 4). The formulation of the reference polyester (P40) has a photo initiator activated by ultraviolet light that triggers the curing reaction, so the addition of an initiator is not necessary (16). However, the P40 had to be protected from UV light during handling and impregnation.

**Table 3:** Reactive polyester/chemical initiator mixture for impregnation and filling of flat chambers.

Group	Preparation	
	Impregnation mixture	Filling mixture
P40	P40 only	P40 only
P18	P18 + 0.125% m/m Butanox^®^	P18 + 0.875% m/m Butanox^®^
C1	C1 only	C1 + 1% m/m Butanox^®^
C2	C2 only	C2 + 1% m/m Butanox^®^
C3	C3 only	C3 + 1% m/m Butanox^®^

Then, a Busch vacuum pump (model R5/0612, air flow 12 m^3^/h, USA) was turned on for five minutes before starting impregnation, so that it reached working temperature (7). Impregnation was started by applying vacuum in the chamber and gradually reducing the pressure (from atmosphere to 5 mmHg). Bubble production began at around 300 mmHg. A high production of acetone bubbles was maintained on the surface of the impregnation mixture. The impregnation process was carried out at room temperature (23±2°C).

Pressure was reduced over a period of 10 h by slowly closing the needle valves until a pressure of 5 mmHg to assure impregnation was complete. The pressure reduction was constant and gradual, as described by Henry and Latorre (16). Impregnation was considered complete when 5 mmHg of pressure was reached, and bubble formation had slowed significantly (16). Impregnation lasted 10 h, the pump was turned off, and the pressure inside the chamber returned to atmospheric and the first 1/3 of the slices (or 25 slices) were ready to plate. The three vacuum runs each contained the 5 polyesters with 5 slices each, totaling 75 slices, and each run was performed under the same conditions and standards. After impregnation, flat chambers were assembled and an impregnated specimen was inserted, filled with polyester mixture, and allowed to cure. Each flat chamber was built using two domestic glass plates (3×20×25 mm) separated by a 6 mm silicone gasket/tubing and secured around the perimeter with metal clips (16).

**Figure 1:**
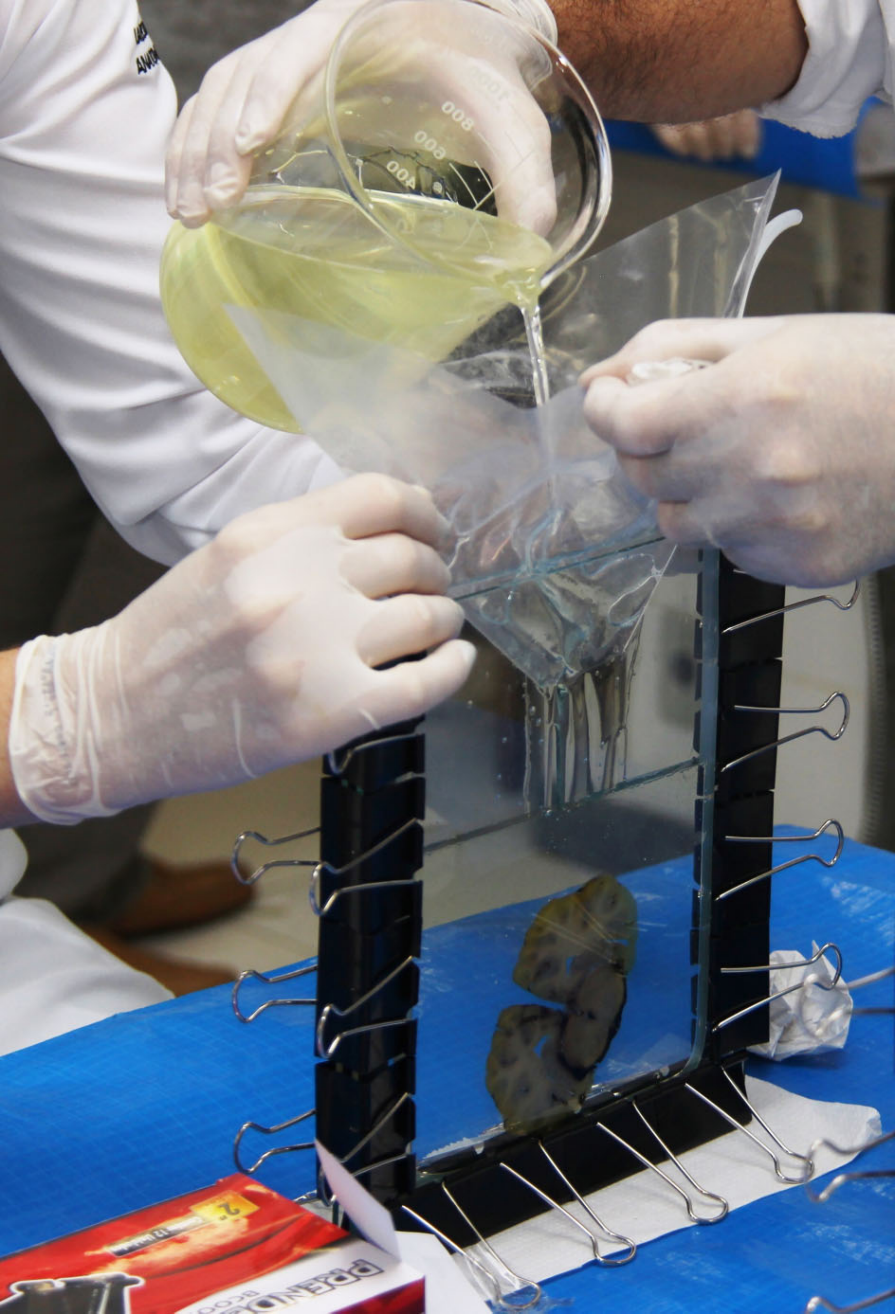
Section positioning and filling of the flat chamber with the polyester mixture.

During flat chamber assembly, the silicone gasket separating the two glass panes was positioned 2 cm from the edge (allowing the clamp to rest over the gasket to prevent resin leakage), creating a “glass × silicone cord × glass” sandwich held together by metal clips. One side was left open until the chamber was filled with the specimen and resin mixture. Each assembled flat chamber was filled with an impregnated slice and the corresponding impregnation resin and initiator (Table 3 and Figure 1). The P18 polyester impregnation mixture already contained a fraction of needed initiator (0.125% v/v). In the next step, the remainder of the initiator was added to complete the 1% (0.875% v/v) amount to the polyester mixture used to fill the flat chamber and consequential curing.

The vertical positioning of the flat chamber allowed air bubbles in the filling mixture to rise to the surface and then be removed using a small syringe with a hypodermic needle. After this procedure, the chamber was closed with the silicone gasket and metal clips.

The assembled flat chambers containing P40 were cured under UV light, whereas specimens impregnated with domestic polyesters were cured without UV light. After 24 h of initial curing, the latter were placed in a 40°C oven for 48 h to accelerate full cure. Since both the photochemical and chemical curing processes are highly exothermic, fans were used for temperature control (16).

After curing, the flat chamber was dismantled, and the finished specimen was removed. This was done by removing the metal clips and using the tip of a scalpel blade to aid in detachment at the junction of the polyester plate and the glass (7).

The suitability of the domestic polyester for plastination of nervous tissue and the quality of the final specimen were verified. The parameters for verifying resin suitability/compatibility included: resin-mix viscosity as a function of impregnation time, behavior of the resin-mix in relation to the forced impregnation steps, curing and disassembly of the flat chambers, transparency of the cured polymers, and specimen shrinkage. Shrinkage was assessed by measuring and recording the percentage shrinkage of brain sections. To evaluate the final specimen, the final stiffness of the cured polyester slices (not malleable), differentiation of white and gray matter, and visual transparency were qualitatively compared with P40 and recorded.

Shrinkage is best calculated by volume measurement (14), which is not feasible in the case of polyester since the sections remain embedded in the plates after impregnation. Thus, the percentage of surface shrinkage was used as a criterion, as shown in Equation 1. This step was performed in triplicate.

$$\begin{array}{l} \frac{area(cm^{2})beforeimpregnation-area(cm^{2})aftercuring}{area(cm^{2})beforeimpregnation}\times 100\\ =shrinkage\% \end{array}$$ (Eq. 1)

To standardize analysis, all sections were identified and photographed immediately after dehydration and after curing. A scale tray was used to hold the camera at a predetermined angle and focal length.

The total surface area of the top of the section was measured using the free software ImageJ (USA), which calculates the area from the number of pixels in the photograph, using a specific scale as a parameter.

In the homoscedasticity analysis of all data sets, statistical assumptions were evaluated by the Levene's test; the Kolmogorov-Smirnov test was used to evaluate the normality of all scalar variables to determine the subsequent statistical tests. A Wilcoxon test was performed to indicate possible differences in section shrinkage within the same group, considering the area before impregnation and after curing. Comparisons between two different groups were performed by the *t*-test for independent samples. A P<0.05 significance level was used in all tests. Calculations and statistical analysis were performed using IBM^®^ SPSS^®^ version 26.0 (USA).

For initiator concentration as a function of curing time test, the coefficient of determination (R^2^) was calculated using Microsoft Excel 2019 (Microsoft Office System 2019, USA).

## Results and Discussion

The standard P40 impregnation protocol recommends a final vacuum of 10 mmHg, since the resin diluent (styrene) is extracted at this pressure. Styrene is known to damage vacuum pumps over time (9). However, there are no established protocols for the tested domestic polyesters and, from that, it was decided to reduce the pressure further to guarantee acetone extraction and complete resin impregnation.

Curing time required for each domestic polyester with Butanox initiator at different concentrations was observed and recorded (Table 4). Only the Redelease (P18) polyester/initiator mixture (0.125%) remained liquid enough to allow impregnation and plating of the slices. None of the Novapol (C's) mixtures remained fluid long enough for impregnation.

**Table 4:** Full curing time by initiator concentration for each sample.

Resin	Sample	Initiator concentration % (m/m)	Time (min)
C1	1	1	14
	2	0.5	16
	3	0.25	24
	4	0.125	56
	5	0.0625	128
C2	6	1	20
	7	0.5	33
	8	0.25	49
	9	0.125	68
	10	0.0625	125
C3	11	1	23
	12	0.5	43
	13	0.25	76
	14	0.125	99
	15	0.0625	121
P18	16	1	23
	17	0.5	48
	18	0.25	118
	19	0.125	2880 (48 h)
	20	0.0625	4560 (76 h)

C1, C2, C3: Cristalan.

Slice impregnation should be completed within 48 h, so that the impregnation mixture must be in a liquid state for casting. Of the domestic polyesters tested, only the P18 polyester sample with 0.125% (m/m) initiator met these requirements: liquid at end of impregnation and curing after 48 h. Therefore, this initiator concentration was chosen for the impregnation mixture for the P18 polymer. Furthermore, no increase in viscosity was noticed in the impregnation mixture after 10 h of impregnation. This occurred because the polymer only enters a gel state (sudden increase in viscosity) closer to the complete curing time.

The Novapol resin mixture samples cured within minutes to a few hours; from an extrapolation calculation, the amount of initiator needed to keep the polymer fluid for an optimum period of time (long enough to thoroughly impregnate the tissue) was too small even for a volumetric pipette, making it unfeasible to measure for mixing into the polymer.

Given these results, the Novapol samples failed to achieve satisfactory results under the circumstances evaluated in this assay. Thus, it was unfeasible to add the initiator in the impregnation step, as was done with P18, restricting its use to the curing stage. Therefore, no initiator was used in the Novapol (C's) impregnation batches.

**Figure 2:**
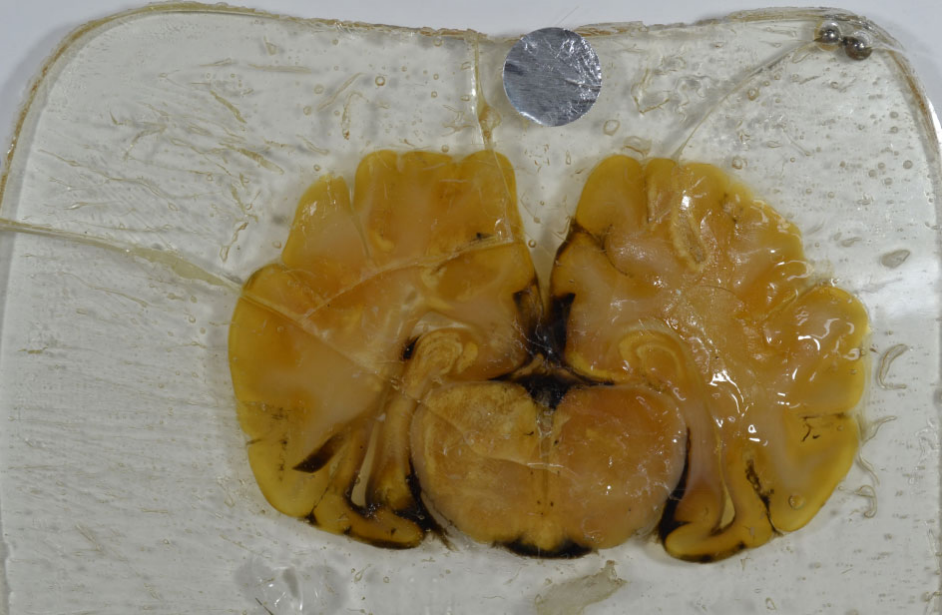
Specimen plastinated with C3 resin showing irregularities and cracks created during disassembling of the chamber.

**Figure 3:**
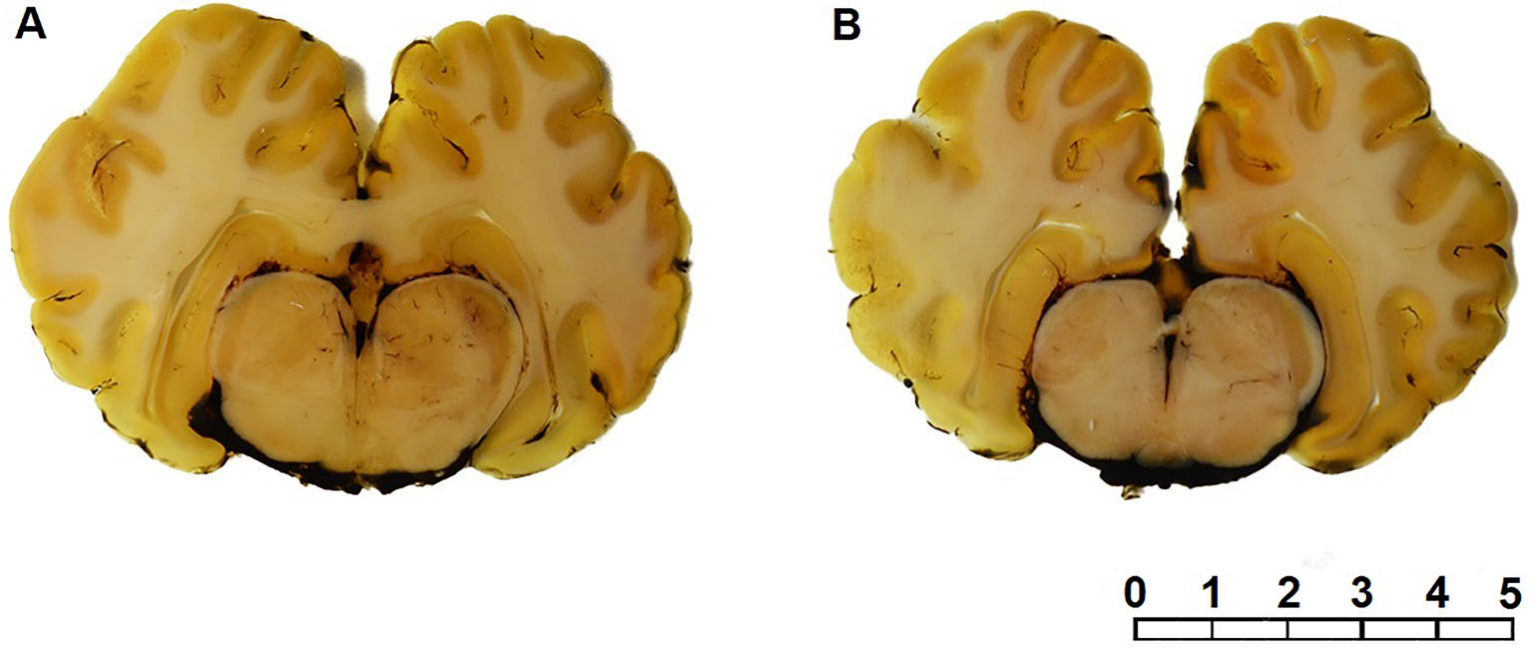
Coronal sections of plastinated brain. **A**, P40 resin and (**B**) P18 resin. Bar=5 cm.

After curing, the flat chambers were dismantled to obtain the finished sections. Sections impregnated with Novapol resins (C1, C2, and C3) were not satisfactory. When disassembling the flat chambers, the resin adhered to the glass plates at various points, preventing slice removal and occasionally leading to breakage of the final specimen and the chamber glass (Figure 2). The points of greatest adhesion were the places where the brain sections touched the glass leaving an uneven surface (Figure 2). This may have been due to the strong adhesion of the polyester without initiator in the tissue to the glass, between the unsaturated polyester resin and the silica in the glass. Thus, subsequent evaluations were discontinued for samples C1, C2, and C3. In turn, the sections plastinated with the reference polyester P40 and P18 were easily removed from the flat chambers (Figure 3).

Mechanical and optical properties of the final specimens (impregnated with P18, domestic polyester) seemed to be as transparent and stiff as the reference polymer (P40). The visual differentiation between the white and gray matter was also excellent. When touching the sections, no wet or sticky areas were noted, which indicated a complete cure in both P40 and P18 specimens. This suggested that the latter may be a substitute for P40.

Tissue shrinkage is an important factor in plastination studies. Regardless of the polymer used (silicone, epoxy, or polyester), the process generates some shrinkage, which is a slight drawback of the technique. Considerable shrinkage can distort the initial shape of the specimen, which is essential for morphometric measurements and imaging comparison (11).

Among the several factors affecting shrinkage, von Hagens (17) and Brown et al. (14) highlight two: 1) dehydration at room temperature from the outflow of water molecules, creating voids and consequently accentuating shrinkage. In this study, this effect was mitigated as dehydration was conducted at low temperatures; and 2) the approximation of molecules during cross-linking, increasing density (18). Unsaturated polyester resins suffer shrinkage of around 5-8% during curing (18).

To verify shrinkage of sections after plastination, the area (cm^2^) of each section was measured after dehydration (immediately before impregnation) and after curing. Table 4 presents the values obtained for each experimental group. The percentage shrinkage rates (PSR) were also calculated from the percentage difference in area (cm^2^) (Table 5).

**Table 5:** Areas of sections before and after impregnation and percentage shrinkage rates (PSR) of P18 and P40.

	P18			P40			
Section	Before (cm^2^)	After (cm^2^)	PSR (%)	Section	Before (cm^2^)	After (cm^2^)	PSR (%)
1	56.71	50.63	10.72	1	37.68	35.02	7.059
2	54.06	52.67	2.575	2	36.90	35.09	4.905
3	51.23	50.53	1.374	3	34.75	32.55	6.331
4	37.00	35.11	5.108	4	37.07	34.84	6.029
5	58.02	54.35	6.325	5	38.68	35.23	8.919
6	57.13	52.15	8.717	6	52.34	48.61	7.126
7	39.30	33.96	13.59	7	35.96	29.37	18.326
8	52.40	46.39	11.47	8	37.27	32.59	12.557
9	45.80	40.73	11.07	9	35.98	30.22	16.009
10	48.22	43.68	9.415	10	34.81	29.37	15.628
11	35.07	31.04	11.49	11	33.48	31.87	4.809
12	36.99	31.85	13.90	12	49.06	43.97	10.375
13	58.70	57.31	2.368	13	58.52	52.99	9.45
14	57.34	54.50	4.953	14	56.05	46.06	17.823
15	33.71	30.32	10.06	15	44.28	40.91	7.602
Mean	48.11	44.35	8.209	Mean	41.52	37.25	10.20
Standard deviation	9.342	9.710	4.129	Standard deviation	8.370	7.459	4.711

Size differed between the impregnated sections depending on the position of the brain: some were more rostral (anterior) and some more caudal (posterior). However, this difference did not influence the shrinkage calculation, since the statistical analyzes did not show a significant difference between the initial volumes of the groups (normality and homogeneity) because sections were randomly distributed into groups.

**Figure 4:**
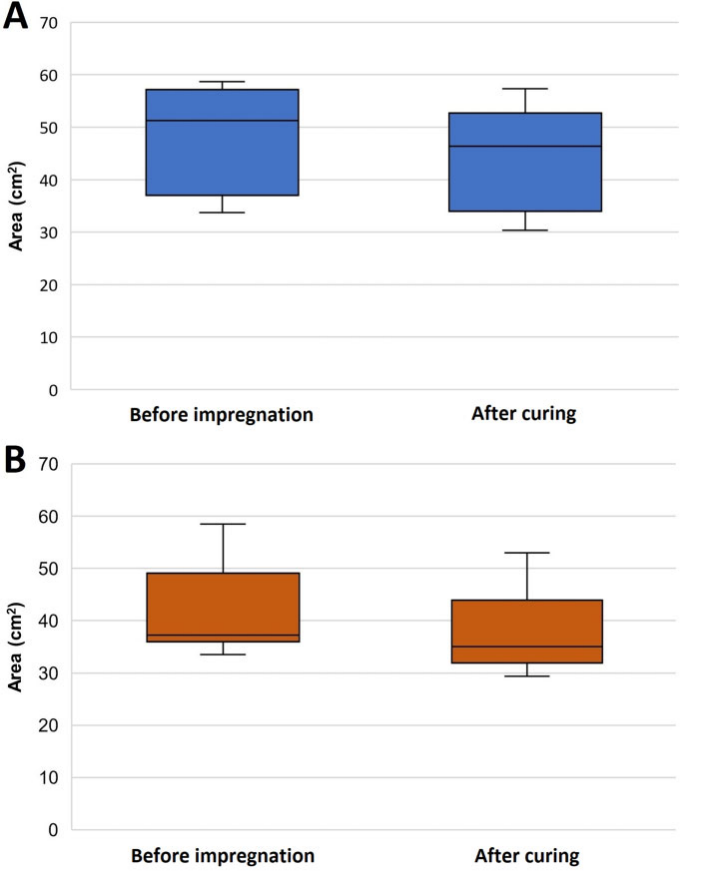
Area of sections before forced impregnation with P18 resin (**A**) and with P40 resin (**B**) and after curing. Data are reported as median and interquartile range.

The area of the sections immediately before impregnation (after dehydration) and after curing were statistically different for both groups analyzed (P=0.001), which showed a shrinkage during plastination (Figure 4), as expected.

When comparing the reference polyester (P40) to the alternative (P18) under the testing conditions, the PSR showed no significant difference (*t*=1.229, df=28, P=0.229) (Figure 4), which confirms our null hypothesis that the mean PSR are equal between the two resins and indicates that the P18 resin can be an alternative to the reference polyester.

**Figure 5:**
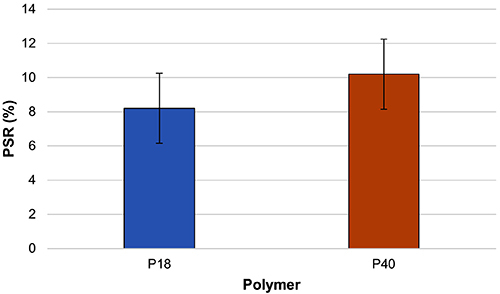
Mean percentage shrinkage rate (PSR) of sections impregnated with the tested polyester resins P18 and P40 (P>0.05, Student’s *t*-test).

Despite the great disparity between viscosities of the polyesters used in this research (Table 1), the tissue shrinkage of the slices impregnated with P40 and P18 were similar (Figure 5). The fact that the slices were thin (2-3 mm) and therefore probably easy to impregnate may not minimize the influence of the different viscosities of the P18 and P40 polyesters on shrinkage. For polyester plastination, a shrinkage rate below 15% as cited by von Hagens (7,17) is considered satisfactory. The comparable PSR of P18 and P40 makes P18 a promising domestic alternative for a polyester mixture that produces good specimens. The results also validated this plastination procedure.

De-bureaucratizing and cost reduction of the technique by using domestically marketed polymers would help disseminate the technique in Brazil, providing numerous advantages to teaching, research, and university outreach. As discussed, plastination eliminates the need to maintain specimens in toxic preservative solutions. Moreover, only plastination allows research on 2-3 mm sections for a long period of time, as it produces sturdy specimens that can be manipulated for observation from all angles. Other preservation methods produce extremely fragile and brittle specimens. In neuroanatomy, for example, brain structures themselves are extremely brittle, overly complex, and closely spaced, leading to the need for thin specimens for demonstration so that information is not lost (19). Plastinated sectioned specimens provide an invaluable bridge between cadavers and radiographic images, as the use of images allows students to work independently and sequentially on a spatial reconstruction (20).

### Conclusions

Of the domestic polyesters examined, only P18 resin showed no significant difference compared to the reference resin (P40) regarding tissue shrinkage. Visually, color and appearance of the final specimens impregnated with P18 were similar to those plastinated with P40 (qualitative visualization).

P18 resin plastination resulted in high quality material, allowing visualization and practical handling. P18 was chosen because of its “plastinic” properties: colorless, rigidity, durability, easy handling, and lower cost compared to the reference polyester. Thus, P18 polyester proved to be a viable alternative to P40, with excellent visual results.

Good reproducibility as well as the good preservation of the slices and their durability are the main qualities for their use in museums, in the teaching of human anatomy, and for comparison with imaging techniques. Compared with other artificial anatomical models, plastinates stand out for their accurate representation of anatomical structures.
